# The effect of health consciousness on older adults’ health information-sharing intention: the mediating role of self-efficacy and social norms

**DOI:** 10.3389/fpubh.2025.1621866

**Published:** 2025-10-14

**Authors:** Jing An, Ziyue Xiang, Kexin Wan, Xuanyu Zhu, Jinlong An, Yujie Yang

**Affiliations:** ^1^School of Management, Nanjing University of Posts and Telecommunications, Nanjing, Jiangsu, China; ^2^The Fourth People’s Hospital of Shenzhen, Shenzhen, Guangdong, China; ^3^First People’s Hospital of Changshu City, Hospital Affiliated to Soochow University, Changshu, Jiangsu, China

**Keywords:** older adults, health consciousness, health information-sharing intention, self-efficacy, social norms

## Abstract

**Background:**

In the modern society where social media and aging are intertwined, how to promote older adults to integrate into the digital society and enjoy digital interests, to improve their quality of life and happiness, and promote active healthy aging has become an important topic today.

**Methods:**

Based on social cognition theory, we constructed a research model including health consciousness, self-efficacy, social norms, and health information-sharing intention (ISI) of older adults. A total of 225 valid questionnaires were collected through a questionnaire survey, and empirical analysis including reliability test, exploratory factor analysis, confirmatory factor analysis, hypothesis test, and mediation effect test was conducted.

**Results:**

The results show that older adults’ health consciousness, self-efficacy, and social norms have a significant positive effect on health information sharing. Health consciousness has a positive effect on social norms and older adults’ self-efficacy. Furthermore, social norms and older adults’ self-efficacy mediate the relationship between health consciousness and health information-sharing intention, respectively.

**Conclusion:**

This study considers health consciousness as a key research variable, which not only further explores the internal drivers of sharing health information among older adults but also provides a new perspective and strategies for promoting healthy aging.

## 1 Introduction

According to data released by the end of 2024, the population aged 60 and above reached 310.31 million, accounting for 22.0% of the China’s total population. Among them, the number of people aged 65 and above in China reached as high as 220.23 million, accounting for 15.6% of the total population, which highlights the continuously high older population and the obvious aging trend. Therefore, in recent years, scholars have paid more attention to older adults, a network user group. In today’s digital society, people often use social media to share their knowledge, insights, and opinions (1, 2), including economic, political, and health information. The use of social media has also become a normal part of life for older adults. For example, among older adults aged 50–65 years in the United States, older adults using social networking sites account for 64% (3). Social media can increase social support and social contact to reduce loneliness in older groups (4), but it also can overcome distance barriers, re-establish and strengthen contacts with friends and relatives, and promote intergenerational communication (5). In addition, in terms of self-cognition, studies have shown that social media can improve self-efficacy and buffer the decline of cognitive ability (6). Therefore, with the rapid development of social media, the focus of users’ information-sharing research has gradually shifted to social media platforms. Research has shown that older adults can improve life satisfaction by using social media (7), reduce loneliness, and improve social life (8). However, due to their auditory and visual cognitive decline, the proportion of older adults using the Internet is still low, and there is a significant “digital divide.” Moreover, older adults’ lack of confidence in technology use may lead to technical anxiety, while privacy concerns (9) and social influence factors (10, 11) may also significantly affect their intention to share information via the Internet or social media. To reduce the digital divide, understanding the factors influencing Chinese older adults’ health information-sharing intentions on social media becomes particularly crucial.

Currently, the research on information-sharing intention (ISI) of older adults mainly focuses on the influencing factors. Through literature sorting, the factors affecting the information sharing of older adults are mainly divided into two categories: individual cognitive factors and external environmental factors. Among them, many scholars argue that individual cognitive factors are crucial in influencing older adults’ intention to share information. In addition to older adults’ information-sharing motivation such as pleasure (8), self-efficacy (12), technology anxiety, and privacy risks (13), studies have also found that older adults can obtain the meaning of life, participation, and contribution to society through sharing information and knowledge online (13, 14).

In terms of the external environment, many studies suggest that the availability of social media platforms, social networking sites, and their content constitutes significant factors encouraging older adults to use such platforms or sites and share information. These factors include perceived ease of use, perceived usefulness, and content quality (13). Meanwhile, social factors have been widely demonstrated to play a critical role in shaping the intention to share information and serve as a significant motivation for older adults to share information. Some scholars believe that the purpose of obtaining health information is mainly for relationship maintenance, and they are willing to share health information with friends and relatives (15). In addition to social relations, which affect the intention to share information, there are also social norms, subjective norms, social support, social pressure, and other factors, that is, important people around them think that older adults should or should not do some behavior (16), thus affecting the will and behavior of older adults. Many studies focus on the impact of social interaction (17) on information sharing among older people, who use social media to contact or interact with family and friends or maintain and expand social contacts, which is an important motivation for older people to use social media and generate intention to share. However, the existing studies lack the effects from other important perspectives, such as health consciousness and health concerns, which may play an important role in influencing the intention of older adults to share health information and deserve further in-depth research. In the context of the aging society, it is important to explore the factors influencing older adults’ intention to share information. Understanding the influencing factors and paths of older adults’ intention to share information can help to bridge the gap between older adults and the digital society and promote older adults to better integrate into the digital age and enjoy the convenience and wellbeing brought by information technology.

Existing studies have laid a solid foundation for understanding older adults’ information-sharing intention, covering key individual cognitive factors (e.g., self-efficacy) and external environmental factors (e.g., social norms). However, there are still three notable gaps that limit their explanatory power for older adults’ health information-sharing behavior. Above all, the majority of studies focus on general rather than health-specific information sharing and may ignore health consciousness—a cognitive factor inherently associated with health-related decision-making—as a key antecedent variable. This oversight may result in the inability to capture the unique motivational logic of health information sharing. Compared with general pleasure or social interaction, older adults’ attention to health issues is more likely to drive their information-sharing intention. Second, existing studies have insufficiently explored the mediating mechanisms between antecedent factors and health information-sharing intention. For instance, it remains unclear whether social norms or self-efficacy play a mediating role between health consciousness and older adults’ health information-sharing intention, which further limits the in-depth understanding of the driving mechanisms behind this behavior. Third, most existing studies examine individual cognitive factors (e.g., self-efficacy) or external environmental factors (e.g., social norms) in isolation, lacking an integrated theoretical framework that connects these factors with older adults’ information-sharing intention.

Existing research on older adults’ technology use and information behavior mainly relies on theoretical frameworks such as the technology acceptance model (TAM), the unified theory of acceptance and use of technology (UTAUT), and social cognitive theory (SCT). Among them, TAM (18) primarily explains technology acceptance through individuals’ perceptions of technology (e.g., perceived ease of use, perceived usefulness, and attitude) but fails to incorporate specific health cognitive factors (e.g., health consciousness) and social environmental influences (e.g., social norms)—both of which are crucial for understanding older adults’ health information sharing rather than mere technology use. Although UTAUT expands on TAM by incorporating factors such as performance expectancy and effort expectancy (19), it fails to comprehensively consider the interactive relationship between individual cognitive factors and environmental factors. In contrast, SCT emphasizes the dynamic triadic interaction between individual cognition, environmental factors, and behavior, providing a solid basis for linking individual cognitive factors and social environmental factors. It not only integrates health consciousness and self-efficacy (individual cognitive factors) as well as social norms (environmental factors) to explain health ISI but also supports the exploration of mediating mechanisms. Therefore, compared with those theoretical frameworks that prioritize individual cognition while ignoring health and social dynamics, SCT is more capable of accurately capturing the complexity of older adults’ health information-sharing behavior in the context of social media.

Therefore, grounded in social cognitive theory, this study adopts a dual perspective encompassing both external environment factors [e.g., social norms (SN)] and individual cognition factors [e.g., health consciousness (HC), self-efficacy (SE)], systematically examining the mechanisms influencing Chinese older adults’ health information-sharing intention in the context of social media. Following standardized scale development procedures, the study draws upon well-established scales, including SN, HC, SE, and ISI, to design the survey questionnaire. Data were collected through questionnaire surveys. For data analysis, descriptive statistics and reliability/validity tests were conducted using SPSS 25.0, while structural equation modeling (SEM) was performed via AMOS 25.0 to validate the path relationships among latent variables. This research aimed to uncover the underlying mechanisms of Chinese older adults’ health information-sharing intention in social media environments, providing both theoretical insights and practical implications for enhancing health information dissemination and optimizing health information services for the older population.

## 2 Theoretical basis and research hypotheses

### 2.1 Social cognitive theory

Albert Bandura’s Social Cognitive Theory was introduced in his book Social Foundations of Thought and Action: A social cognitive theory (1986) (20). The book emphasizes that human activities are not determined by a single factor but are the result of the interweaving and interaction between individual behavior, individual cognition, and their environment (21, 22). This triadic reciprocal determinism constitutes the core framework of social cognitive theory. This triadic reciprocal model can be decomposed into three sets of reciprocal relationships (23): First, individual cognition controls and drives one’s behavior, while behavior, in turn, influences cognition through internal reflection and external feedback; second, environmental factors stimulate and shape individual cognition, and at the same time, individuals can subjectively perceive, interpret, and grasp environmental factors (24) through their self-efficacy, and third, environmental factors determine the direction and intensity of behavior, while behavior itself also can transform the environment (25). It should be noted that in scenarios combining different individuals and different environments, the relative influence exerted by these three sets of interactive factors varies (20). The triadic interaction model is characterized by two key features: “asymmetry of influence” and “temporal asymmetry in effects,” which make it possible for us to conduct targeted decomposition and analysis of the model. Given that this study focuses on the influencing mechanism of older adults’ information-sharing intention in the context of social media and incorporates health consciousness as a core variable, we selected three influence paths for exploration, namely “Cognitive Factors → Behaviors”, “Cognitive Factors → Environmental Factors”, and “Environmental Factors → Behaviors”. Specifically, these paths include the following: the direct influence of individual cognition on environmental factors and behavior, the direct influence of environmental factors on behavior, and the indirect influence of individual cognition on behavior through environmental factors. In summary, based on social cognitive theory, this study defines social norms (SN) as an environmental factor and health consciousness (HC) and self-Efficacy (SE) as individual cognitive factors to systematically analyze the influencing mechanism of older adults’ information-sharing intention in the context of social media.

### 2.2 Research hypotheses

#### 2.2.1 Health consciousness

Health consciousness is a tendency to focus on health, part of consciousness, and expresses the degree of individual involvement in health problems or willingness to engage in various health behaviors (26), including knowledge about health, interest in health issues, tendency to seek health information and personal health action, which includes prevention and attention to health, adherence to the health action lifestyle, establishment of social relations and support systems, and health information-seeking skills (27). As an individual’s cognition and attitude, health consciousness is particularly important for older adults. With the decline in physical functions, older adults tend to attach greater importance to health knowledge and health maintenance, and this health consciousness prompts them to take a series of positive behaviors to preserve their health. These behaviors not only include adjustments to personal living habits but also may involve interactions with others and information sharing. Researchers find that personal health consciousness has a direct impact on the use of health applications and information (28). Health information will induce different cognitive responses according to the level of health consciousness (29). People with higher health consciousness may find the information more relevant to individuals, pay more attention to the information, and may be more systematic in thinking about the arguments and recommendations contained in the information (30). There is evidence that people with high levels of health consciousness tend to have a healthier lifestyle (29). With the decline of body function, older adults will pay more attention to absorbing health-related knowledge to maintain their health, as health consciousness will affect the attitudes toward physical activity (31). Health consciousness may cause older adults to take a series of positive behaviors to maintain their health, including sharing their experience, knowledge, and suggestions related to health on social media, which may improve their confidence and make them more confident that they can provide valuable information and help other users to solve the problem. Meanwhile, when older adults possess a high level of health consciousness, they are more likely to become advocates and practitioners of healthy behaviors. Their behaviors, such as paying attention to and sharing health information, engaging in regular physical exercise, and focusing on dietary adjustments, can generate a certain demonstration effect in society. As more and more older adults exhibit a strong sense of health consciousness and adopt positive health behaviors, these behaviors will become more prevalent and widespread in society. Gradually, these behaviors will form a norm in society (32), that is, people around them, including family members, friends, neighbors, etc., will be affected by these social norms to pay attention to health information and participate in health-related activities. Based on the above analysis, the following assumptions are made:

*H1:* The health consciousness of older adults has a positive impact on social norms.

*H2:* The health consciousness of older adults has a positive impact on self-efficacy.

Previous studies have revealed that health information-sharing behaviors of social media users are influenced by multiple factors such as value, environment, and individual cognition, which include subjective norms, self-efficacy, behavioral intentions, and health literacy (33). Meanwhile, there are some studies indicating a connection between health consciousness and health literacy (34). Health literacy is closely related to people’s health information-sharing behaviors in the family (35). In summary, health consciousness is related to individuals’ information-sharing behaviors. There are also studies demonstrating that individuals with higher levels of health consciousness have a better ability to search, discover, critically reflect, and apply health-related information (29). Based on the above analysis, the following assumptions are proposed:

*H3:* The health consciousness of older adults has a positive impact on health information-sharing intention.

#### 2.2.2 Self-efficacy

Self-efficacy refers to the judgment of individuals about their ability to perform a specific task (36), which is defined in this study as the degree of confidence of older adults in their ability to share information on social media. Wu et al. (33) drew on the theory of planning behavior, use and gratifications theory, and social cognition theory, with the help of structural equation model analysis, and found that perceived self-efficacy can affect users’ health information-sharing behavior on WeChat. Furthermore, it shows that increased self-efficacy can motivate knowledge contributors to share knowledge with others (37), and that self-efficacy also influences the intention of Facebook users to share information (1). In the context of social media, intrinsic motivation can enhance information-sharing behavior (38) when people experience increased self-efficacy by using and experiencing social media.

*H4:* The self-efficacy of older adults has a positive impact on health information-sharing intention.

Considering the existing discussions on the impact of health consciousness on self-efficacy and the influence of self-efficacy on the intention to share information, this study infers that self-efficacy mediates the relationship between health consciousness and the intention to share information among older adults. For example, in addition to the direct and significant effects of self-efficacy on information sharing, self-efficacy also plays a mediator between self-connectivity and social media information sharing (39). Another study explained the mediation role of self-efficacy on the relationship between social support and resilience (40). Self-efficacy also plays a mediating role in the relationship between health literacy and quality of life in Tibet, China (41). Student self-efficacy can also play a mediating role between the learning environment and self-regulation (42). Based on the above analysis, the following assumptions are made:

*H5:* Self-efficacy in older adults mediates the relationship between health consciousness and information-sharing intention.

#### 2.2.3 Social norms

Cialdini (43) defines social norms as rules and standards understood and endorsed by members of a society, which guide and constrain social behavior in the absence of legal mandates. In addition, the focus theory of normative conduct divides social norms into two categories: descriptive norms, referring to the perceived prevalence or typicality of a behavior within a group, and injunctive norms, referring to the perceived social approval or disapproval of that behavior (32, 44). Synthesizing the above two definitions and aligning with the research focus, this study defines social norm as: the level of recognition and support for information-sharing behavior by the majority of people in society, including family, friends, and important people around them. Social psychology shows that social norms have a great influence on behavior (45). In addition, some studies have proved that in the virtual social environment of social media, the attitudes and behaviors of others will lead to social norms, thus affecting individual behavior (44). Social information processing theory (46) believes that to be effective organization members, employees find information from their social environment, including social norms, values, expectations, and the consequences of their observed behavior, thus helping individuals to act more strategically and choose directly or indirectly to lead to more positive results, because they are consistent with social expectations (32). Similarly, some studies have shown that the attitudes of some reference objects with respect to things will lead to normative pressure, prompting individuals to comply with the attitudes of these reference objects (47). For example, the peer-friend effect will affect Facebook users’ intention to share information (1). Based on the above analysis, the following assumptions are made:

*H6:* Social norms have a positive impact on older adults’ health information-sharing intention.

Based on the above discussion about the influence of health consciousness on social norms and the role of social norms in enhancing older adults’ intention to share information, this study hypothesizes that social norms play a mediating role between health consciousness and the intention of older adults to share information. Previous studies in health communication have provided evidence for the mediating role of social norms. For example, some studies found that social norms play a significant intermediary role between adolescent smoking (48). Social norms play a mediating role in the relationship between social media use and alcohol use (49). Junejo et al. (50) employed PLS-SEM to explore the mediating role of social norms: specifically, its mediation between perceived behavioral control and e-commerce usage intention, as well as between perceived external pressure and e-commerce usage intention, during COVID-19. Based on the above analysis, the following assumptions are made:

*H7:* Social norms play a mediating role in the relationship between health consciousness and information-sharing intention among older adults.

**Figure 1:**
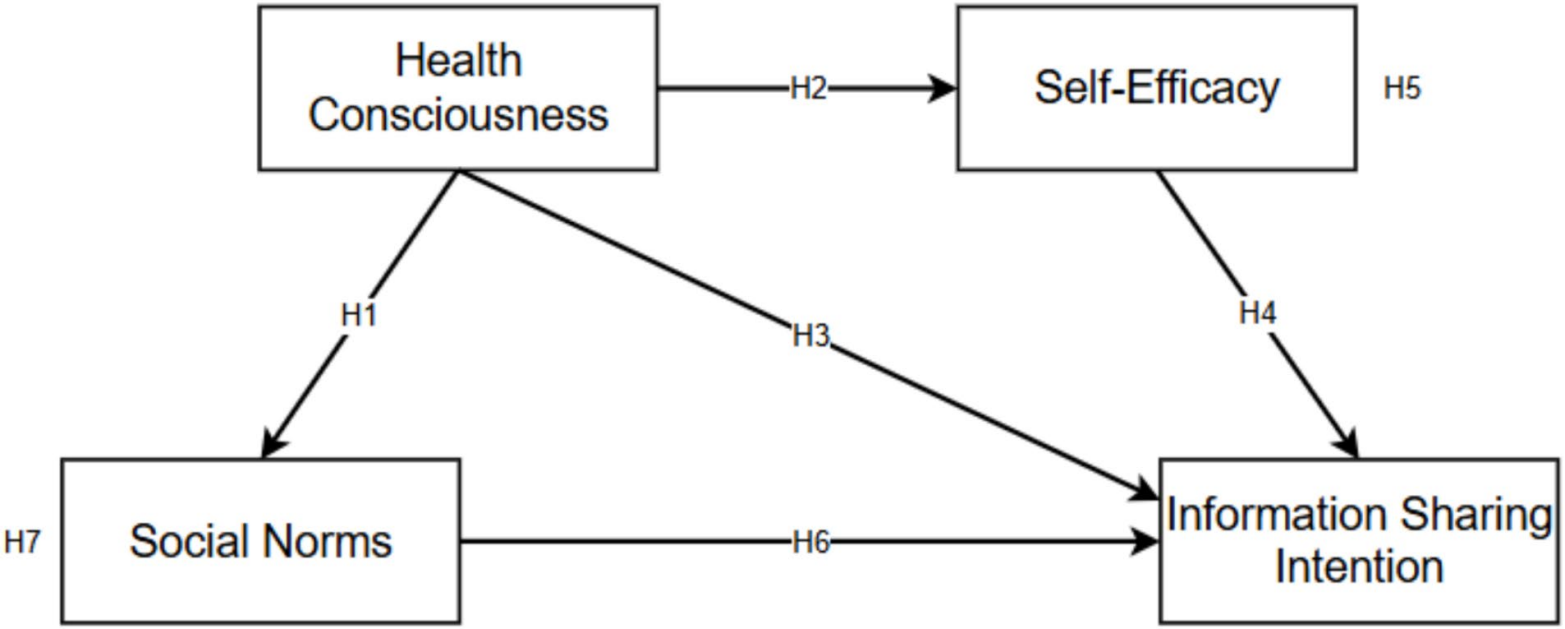
Research model of influencing factors of older adults’ information-sharing intention in social media.

We proposed the research model of this study as follows (Figure 1).

## 3 Research methods

### 3.1 Design

This study used the questionnaire method to systematically design and investigate the factors influencing Chinese older adults’ intentions to share health information on social media platforms. Before the respondents began to fill in the questionnaire, older adults who did not meet the subjects’ requirements were screened by setting the question “Have you used WeChat, TikTok, Weibo, TopBuzz, and REDnote.” The questionnaire contains the basic information and scale items (Table 1). The basic information includes gender, age, education, and occupation before retirement. The scale part contains the measurement items of four variables, such as health consciousness (26), self-efficacy (51), social norms (52), and information-sharing intention (53). All scales use the Likert five-level scale (1 = very disagree, 2 = disagree, 3 = uncertain, 4 = agree, 5 = very agree), reflecting the degree of conformity between the actual situation of the respondents and the description of the questions.

**Table 1:** Measurement items of each variable.

Variable	No.	Items	Source
Health consciousness	HC1	I often pay attention to my health	(26)
	HC2	I will focus on health-related information and products	
	HC3	I exercise a lot	
	HC4	I will try my best to relieve stress and relax the mood	
	HC5	I know more about health than others	
	HC6	I have a responsibility to take good care of myself to prevent disease	
Self-efficacy	SE1	I think I have the ability to provide valuable health information	(51)
	SE2	I am confident that the shared health information can help other users solve their problems	
	SE3	I have shareable health information and experience	
Social norms	SN1	Many people in society have ever had health information-sharing behaviors	(52)
	SN2	Family, friends, and important people around me think I should share health information	
	SN3	Family members, friends, and very important people are positive about health information-sharing behaviors	
Information-sharing intention	ISI1	I will share my health experience and information through WeChat, TikTok, Weibo, TopBuzz, REDnote, and other social media	(53)
	ISI2	When I have health problems, I will send messages to social media platforms for help	
	ISI3	I will answer users’ questions through sharing health information on the social media platform	
	ISI4	I would recommend others to use social media platforms to share their health information	
	ISI5	I strongly agree with the health information-sharing behaviors of users on social media platforms	

### 3.2 Data collection

The research objects of this study were Chinese older adults over 50 years old who had used social media. The questionnaires were used to collect the information of the respondents. The distribution and collection of questionnaires were carried out online and offline from February to April 2023. The distribution of online questionnaires is based on “https://www.wjx.cn/,” an online questionnaire survey platform in China, and the offline collection sites are mainly distributed in some communities and parks with a dense population of older adults in China. A total of 248 questionnaires were collected. In this study, abnormal and invalid samples were excluded, including those with a response time of less than 60 s and those with the same option chosen consecutively for multiple questions. Finally, 225 valid questionnaires were included, which met the minimum sample size required for the study (54), with an effective sample recovery rate of 90.7%.

## 4 Data analysis

### 4.1 Descriptive statistics

A descriptive statistical analysis using SPSS 25.0 software obtained the frequency distribution of the basic characteristics of the samples. From the perspective of gender, the proportion of men (56.4%) and women (43.6%) was relatively balanced. The age was concentrated between 60 and 74 years old. Considering the educational level of the respondents, 93.3% of them had a bachelor’s degree. From the occupation of the respondents before retirement, enterprise employees and private business owners were the majority (Table 2).

**Table 2:** Descriptive statistics of the sample questionnaire data.

Variable	Items	Frequency	Percentage
Gender	Men	127	56.4%
	Women	98	43.6%
Age	50–59 years old	17	7.6%
	60–64 years old	77	34.2%
	65–69 years old	88	39.1%
	70–74 years old	27	12.0%
	75–79 years old	11	4.9%
	Aged 80 and above	5	2.2%
Education	Primary school and below	36	16%
	Junior middle school	81	36%
	Senior middle school	54	24%
	Junior college education	39	17.3%
	Bachelor’s degree or above	15	6.7%
Occupation before retirement	Civil servants or public institution workers	41	18.2%
	Enterprise staff	88	39.1%
	Private business owners	53	23.6%
	Free profession	37	16.4%
	Others	6	2.7%

### 4.2 Reliability test

The scale reliability results show that the Cronbach’s α coefficient corresponding to the four variables is greater than 0.7 (Table 3). In addition, the corrected item-total correlation of each item is more than 0.5, which means that these items have a high correlation with other items within one dimension. The Cronbach’s α coefficient after this item being deleted is less than the Cronbach’s α coefficient of the variable, indicating that the reliability of this dimension is not improved after this item was deleted. In conclusion, the internal consistency of each dimension of the questionnaire is good, indicating that the questionnaire has good reliability and the questionnaire data are reliable, which can be used for subsequent analysis.

**Table 3:** Reliability test results.

Variable	Items	Corrected item-total correlation	Cronbach’s α coefficient after this item being deleted	Cronbach’s α coefficient
Health consciousness	HC1	0.595	0.871	0.877
	HC2	0.712	0.850	
	HC3	0.797	0.836	
	HC4	0.645	0.862	
	HC5	0.762	0.842	
	HC6	0.594	0.870	
Self-efficacy	SE1	0.661	0.766	0.819
	SE2	0.669	0.755	
	SE3	0.692	0.734	
Social norms	SN1	0.661	0.699	0.796
	SN2	0.606	0.761	
	SN3	0.655	0.708	
Information-sharing intention	ISI1	0.704	0.811	0.852
	ISI2	0.638	0.829	
	ISI3	0.636	0.829	
	ISI4	0.746	0.800	
	ISI5	0.599	0.839	

### 4.3 Exploratory factor analysis

Exploratory factor analysis was further performed using the KMO test and Bartlett’s test of sphericity. The KMO value was 0.895 (>0.8), and the significance of the Bartlett’s test of sphericity was less than 0.05, both of which pass the test, indicating the good structural validity of the questionnaire data.

### 4.4 Confirmatory factor analysis

The fit of the model to the data was judged by the goodness-of-fit test. This test usually contains several important observation indicators, such as $\chi^{2}$/df, GFI, RMSEA, RMR, and CFI, and when the model meets the above indicators, the fit of the model is good. According to the evaluation criteria of model fitting indicators, all fitting indices, including absolute fit indices such as RMR and RMSEA, and value-added fit indices such as NFI and TLI, and $\chi^{2}$/df and PGFI, meet the criteria (see Table 4), so the model fit is good.

**Table 4:** Model fitting indicators.

Indicator	Standards	Results	Model adaptation judgment
Absolute fit index			
RMR	<0.05	0.044	Yes
RMSEA	<0.05	0.035	Yes
GFI	>0.9	0.933	Yes
AGFI	>0.9	0.909	Yes
Value-added fix index			
NFI	>0.9	0.923	Yes
RFI	>0.9	0.908	Yes
IFI	>0.9	0.982	Yes
TLI	>0.9	0.978	Yes
CFI	>0.9	0.982	Yes
Minimalist fit index			
PGFI	>0.5	0.689	Yes
PNFI	>0.5	0.767	Yes
PCFI	>0.5	0.816	Yes
$\chi^{2}$/df	<2	1.282	Yes

In the case that the questionnaire scale has a good fit, this study further tests the convergent validity of all dimensions of the scale. The standardized regression weights are greater than 0.6 and significant. In addition, the composite reliability (CR) value of the four variables of health consciousness, self-efficacy, social norms, and information-sharing intention is greater than 0.7. The average variance extracted (AVE) of the variables is greater than 0.5, and the square multiple correlations (SMC) value of all scale questions is not less than 0.36 (Table 5), indicating that the questionnaire scale of this study has good convergent validity.

**Table 5:** Convergent validity.

Variable	Items	Parameter significance estimation				Standardized regression weights	SMC	CR	AVE
		Unstd.	S.E.	*t*-value	*p*				
Health consciousness	HC1	1.060	0.127	8.313	***	0.635	0.403	0.882	0.557
	HC2	1.248	0.128	9.716	***	0.769	0.591		
	HC3	1.317	0.124	10.591	***	0.865	0.748		
	HC4	1.146	0.128	8.988	***	0.697	0.486		
	HC5	1.273	0.123	10.343	***	0.836	0.699		
	HC6	1.000	—	—	—	0.645	0.416		
Self-efficacy	SE1	1.000	—	—	—	0.752	0.566	0.821	0.605
	SE2	0.950	0.092	10.292	***	0.762	0.581		
	SE3	0.982	0.092	10.641	***	0.817	0.667		
Social norms	SN1	1.010	0.096	10.469	***	0.774	0.599	0.798	0.569
	SN2	0.929	0.098	9.509	***	0.683	0.466		
	SN3	1.000	—	—	—	0.802	0.643		
Information sharing intention	ISI1	1.203	0.129	9.353	***	0.762	0.581	0.854	0.540
	ISI2	1.129	0.129	8.775	***	0.701	0.491		
	ISI3	1.128	0.126	8.930	***	0.716	0.513		
	ISI4	1.324	0.132	10.004	***	0.841	0.707		
	ISI5	1.000	—	—	—	0.640	0.410		

***p < 0.001.

A test of discriminant validity was performed. As shown in Table 6, the square root of the AVE value of each variable was greater than the correlation coefficient between the variable and other variables, indicating that the variables were correlated with each other and had good discriminant validity.

**Table 6:** Discriminant validity: Pearson correlation and AVE square root.

Latent variables	Information-sharing intention	Social norms	Self-efficacy	Health consciousness
Information-sharing intention	**0.735**			
Social norms	0.597	**0.754**		
Self-efficacy	0.525	0.327	**0.778**	
Health consciousness	0.561	0.538	0.422	**0.746**

The diagonal is the square root of average variance extracted (AVE).

### 4.5 Path coefficients and hypothesis testing

**Figure 2:**
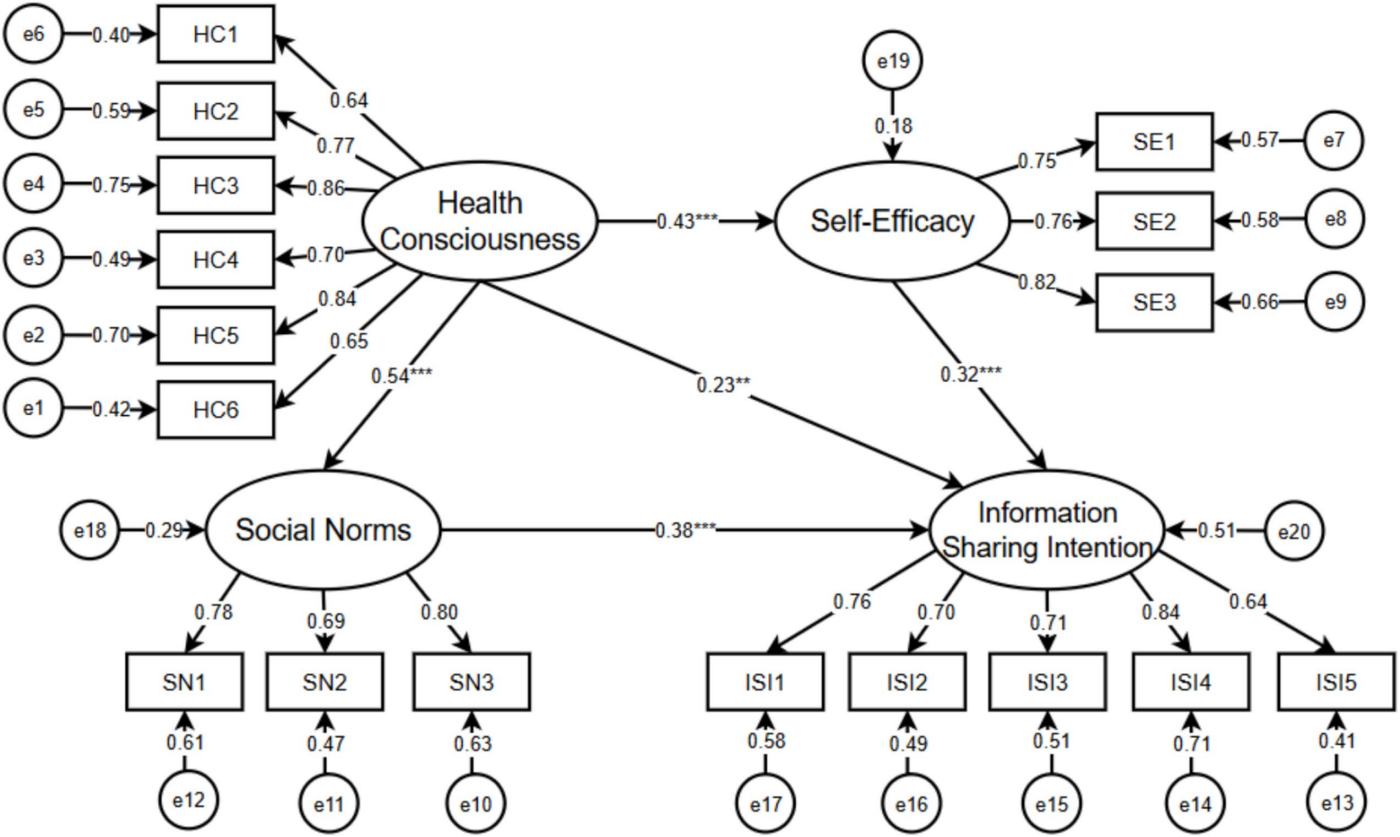
Model diagram of the structural equation.

AMOS 25.0 software was adopted to analyze the path coefficient between each variable to determine the relationship between the variables and the hypothesis results. The *p*-value of each path was less than 0.05, and C.R. values were greater than 1.96 (Table 7), indicating a significant positive effect of health consciousness on social norms and self-efficacy, as well as health consciousness, self-efficacy, and social norms on information-sharing intention. Therefore, hypotheses H1, H2, H3, H4, and H6 are supported. The final results of the structural equation model test are shown in Figure 2.

**Table 7:** Pathway coefficient and hypothesis testing results.

Hypothesis	Way	Standardization coefficientβ	S.E.	C.R.	*p*	Result
H1	Health consciousness → Social norms	0.543	0.105	6.269	***	Support
H2	Health consciousness → Self-efficacy	0.428	0.103	5.103	***	Support
H3	Health consciousness → Information-sharing intention	0.225	0.089	2.596	0.009	Support
H4	Self-efficacy → Information-sharing intention	0.317	0.065	4.076	***	Support
H6	Social norms → Information-sharing intention	0.382	0.075	4.337	***	Support

***p < 0.001.

### 4.6 Mediation effect test

The bootstrap sampling method (55) was used to verify the mediation role of social norms and self-efficacy between health consciousness and information-sharing intention. We performed the 5,000 repeated sampling of the samples at the set 95% percentile confidence level and 95% bias-corrected confidence level and observed the upper and lower limits of the confidence interval for indirect effects. As shown in Table 8, the estimated values of health consciousness indirectly influencing information-sharing intention through social norms and self-efficacy were 0.207 and 0.136, respectively, and both passed the significance test (*p* < 0.05). The indirect effect and direct effect confidence intervals did not include 0, indicating that the mediation effect was significant and the partial mediation effect was proven. To be specific, social norms and self-efficacy partially mediate the relationship between health consciousness and information-sharing intention. Therefore, the hypotheses H5 and H7 are supported.

**Table 8:** Analysis of the mediation effects.

Effect type	Estimate	Bias-corrected 95% C			Percentile 95% CI		
		Lower	Upper	*p*	Lower	Upper	*p*
The direct effect of health consciousness on information-sharing intention	0.225	0.019	0.408	0.036	0.028	0.416	0.030
The indirect effect of health consciousness, social norms, and information-sharing intention	0.207	0.109	0.337	0.000	0.104	0.329	0.000
The indirect effect of health consciousness, self-efficacy, and information-sharing intention	0.136	0.064	0.243	0.000	0.056	0.231	0.001
The overall effect of health consciousness on information-sharing intention	0.568	0.429	0.684	0.000	0.431	0.686	0.000

## 5 Discussion

Against the backdrop of the rapid development of social media platforms such as REDnote, TikTok, and WeChat, coupled with the continuous deepening of population aging, social media has gradually become an important channel for older adults to obtain and share information. As society’s attention to older adults increases, the information-sharing behaviors of older adults have increasingly become a hot topic in the field of information behavior research. How to promote the integration of older adults into the digital society, protect their digital rights and interests, and thereby advance active and healthy aging has become an important issue requiring urgent exploration.

To further investigate the pathways for promoting older adults’ intention to share information in the context of social media, this study constructs a research model of the influencing factors of older adults’ information-sharing intention based on social cognitive theory. In this model, “health consciousness” and “self-efficacy” are classified as individual cognitive factors, “social norms” as environmental factors, and “information-sharing intention” as a behavioral factor. The study collected data through questionnaire surveys and conducted empirical analyses using AMOS 25.0 and SPSS 25.0 software, including descriptive statistics, reliability tests, exploratory factor analysis, confirmatory factor analysis, hypothesis testing, and mediating effect testing. The results showed that the questionnaire data have high reliability and the model has a good fit. The specific research conclusions are as follows: Older adults’ health consciousness, self-efficacy, and social norms all have a significant positive impact on their information-sharing intention. Specifically, the improvement of health consciousness, the enhancement of confidence in their own ability to share information on social media, or the increase in society’s recognition and support for information-sharing behaviors will all promote older adults’ intention to share information. Additionally, older adults’ health consciousness has a positive promoting effect on social norms and self-efficacy. Meanwhile, social norms and self-efficacy play a mediating role between health consciousness and information-sharing intention. This study offers theoretical and practical implications for understanding and promoting older adults’ information-sharing behavior in social media contexts, contributing to the realization of digital inclusion and healthy aging.

### 5.1 Theoretical implications

Against the context of social media, this study conducted an in-depth exploration of the mechanism of action between health consciousness, self-efficacy, social norms of older adults, and their intention to share information. Through empirical analysis, this study found that health consciousness of older adults has a significant positive impact on their self-efficacy, information-sharing intention, and the formation of social norms. Health consciousness demonstrates the most significant influence on social norms related to health information-sharing behaviors. In this study, health consciousness is defined as an individual’s propensity to engage with health-related matters, encompassing health knowledge, interest in health issues, information-seeking tendencies, and proactive health behaviors (26, 27). When older adults develop greater confidence in their ability to share health information through social media platforms, this enhanced health consciousness leads them to perceive stronger social endorsement of such behaviors. More specifically, they become more likely to recognize that health information sharing receives substantial approval from their social environment, including family members, friends, and other significant individuals within their social circles. Especially, the stronger the health consciousness of older adults, the more they can improve their intention to share health information. The results of this study are consistent with the research findings about the impact of health consciousness on the search, processing, and use of health information (29, 34), and that frequent sharing of health information by people around them can lead older people to perceive health information sharing as widespread and highly supported by the general public, which, in turn, has an impact on social norms. Duong et al. (56) found that the information about e-cigarettes on social media was positively correlated with the popularity of e-cigarettes; namely, social media users’ judgments of social norms on e-cigarettes are influenced by relevant information on social media, which is consistent with the mechanism by which health consciousness has a positive impact on social norms. In addition, individuals with high health consciousness tend to actively participate in physical exercise (57) and community-led health activities (58) and become more proficient in health-related knowledge and integrate it into their daily life (59). The accumulation and practice of the knowledge will gradually increase their self-efficacy.

In addition, social norms have a positive impact on the intention of older adults to share health information, consistent with the result that peer friends can affect the information-sharing intention of Facebook users (1). This study also found that the self-efficacy of older adults positively influenced their intention to share health information, in line with previous findings that self-efficacy affected the information-sharing behavior of WeChat users (33). To elucidate the mechanism linking health consciousness with health information-sharing intention, this study examined the mediating roles of social norms and self-efficacy. The findings reveal that both social norms and self-efficacy serve as significant mediators in the relationship between older adults’ health consciousness and their information-sharing intention. Specifically, by strengthening social norms and enhancing self-efficacy, the positive effect of health consciousness on sharing intention can be significantly amplified. This suggests that effective strategies to promote health information sharing among older adults should not only focus on cultivating health consciousness but also on creating supportive social environments and improving their confidence in sharing capabilities. These findings align with existing literature demonstrating the mediating roles of these constructs. For instance, Junejo’s et al. (50) study established social norms as a mediator between perceived behavioral control, external pressures, and e-commerce purchase intentions, illustrating how normative influences operate across different behavioral contexts. Similarly, research has shown that self-efficacy mediates the relationship between self-connectivity and social media information sharing (39), underscoring its fundamental role in communicative behaviors. Although these studies investigated diverse populations and behaviors, they collectively demonstrate the fundamental importance of social norms (as a crucial socio-environmental factor) and self-efficacy (as a key individual cognitive factor) as universal mechanisms influencing behavioral intentions—a conclusion that our study specifically extends to the context of health information sharing among older adults. Consequently, developing interventions that simultaneously enhance supportive social norms surrounding health information sharing and strengthen older adults’ self-efficacy in using social media platforms may prove effective in promoting health information-sharing intentions within aging populations.

### 5.2 Practical implications

Based on the findings of this study, enhancing older adults’ intention to share health information requires a coordinated effort from two aspects: individual cognition and social environment. At the individual cognitive level, it is essential to strengthen health consciousness and self-efficacy, with the aging-friendly transformation of social media platforms serving as a crucial support. This involves integrating health knowledge into community health lectures and interest activities, providing information through age-appropriate health-related apps, and establishing a comprehensive health system covering physical examinations and psychological support to encourage older adults to proactively pay attention to health and accumulate relevant knowledge. Meanwhile, platforms need to optimize interface designs to adapt to the sensory characteristics of older adults, develop health-specific functions, offer digital skills training, and strengthen security protection. Such measures will simplify operations, help older adults master usage skills, and thereby enhance their confidence and ability to share health information via social media. At the social environmental level, it is necessary to foster positive social norms. Family members should take the lead in guiding older adults to share information; communities should organize online and offline activities and establish sharing groups; social organizations should provide skills training; media should publicize positive cases; and the government should introduce supportive policies. These joint efforts will create an atmosphere of universal social support. Through the coordinated advancement of individual and social-level measures, older adults can be better integrated into the digital society, promoting healthy aging.

## 6 Limitations and future research

This study provides ideas for how to improve the intention of Chinese older adults to share health information, to promote the effective interaction and integration between older adults and society, and to enhance their social belonging and happiness. This helps not only relieve their loneliness and social anxiety but also improve their quality of life and social participation. Meanwhile, with the increasingly obvious aging trend, older adults continue to pay more attention to health issues. Given that social media has become the core platform of information dissemination, sharing health information is particularly important for the health management and improvement of older adults. Therefore, considering health consciousness as a key research variable can not only further explore the internal drivers of sharing health information among older adults but also provide a new perspective and strategies for promoting healthy aging. This study has provided meaningful insights into the health information-sharing intention of Chinese older adults while also acknowledging the opportunities for further improvement. The sample size could be enlarged and enriched in future research by incorporating more diverse demographic characteristics and regional differences, especially enlarging the sample size of the very old with aged 70 and above, which will enhance the representativeness and robustness of the research findings. The present model has successfully identified three core determinants of the intention to share. Meanwhile, it is recognized that supplementary factors such as technological anxiety (13) and self-perception of aging (60) may offer a more nuanced understanding of the willingness of older adults to share health information. These potential expansions will contribute to complementing and strengthening the current framework.

## Data Availability

The raw data supporting the conclusions of this article will be made available by the authors, without undue reservation.
